# Supplementary material for: Urban vacant lands impart hydrological benefits across city landscapes
Source: Nat Commun. 2020 Mar 26;11:1563. doi: 10.1038/s41467-020-15376-9 (PMC7099022; doi:10.1038/s41467-020-15376-9)
Supplement: Supplementary file 1 — Supplementary Information [file 41467_2020_15376_MOESM1_ESM.docx]

**Supplementary Information for**

Urban vacant lands impact hydrological benefits across city landscapes

by Kelleher et al.

Corresponding Author: Christa Kelleher

Email: ckellehe@syr.edu

**Supplementary Figures:**


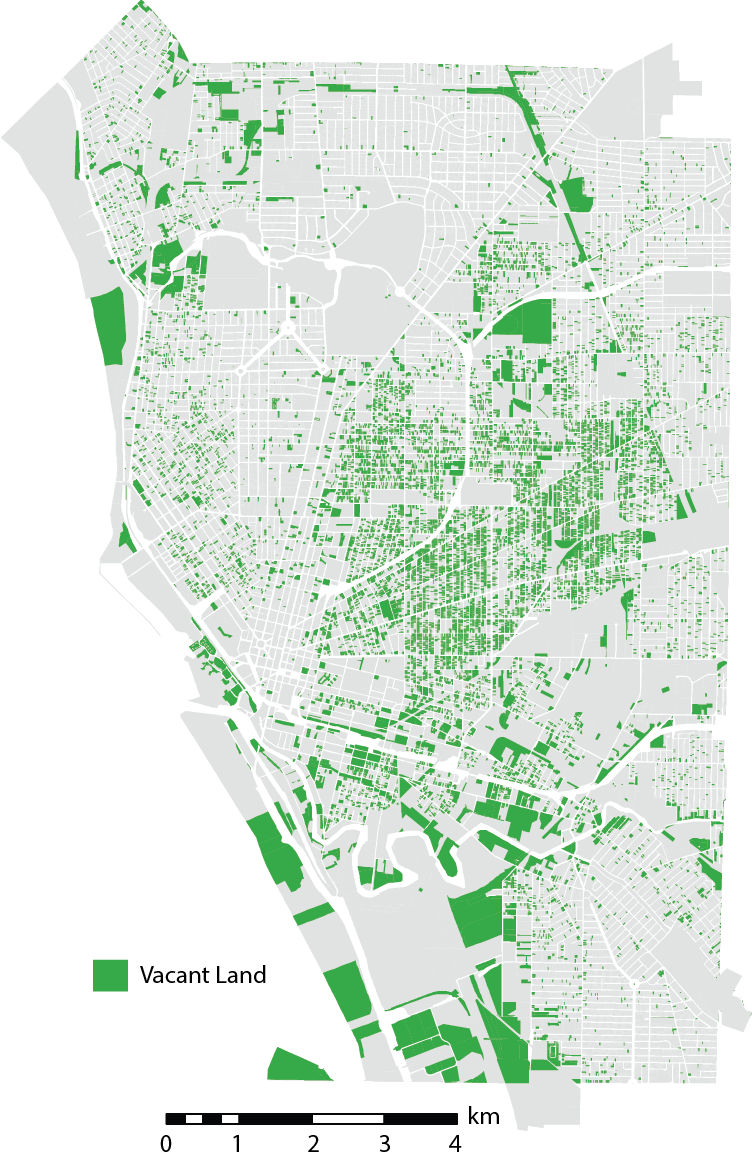


**Supplementary Figure 1:** Vacant parcels across the city of Buffalo (based on data from 2018)^1^. Together, these 17,143 properties represent a cumulative vacant land area of more than 15.1 square km.


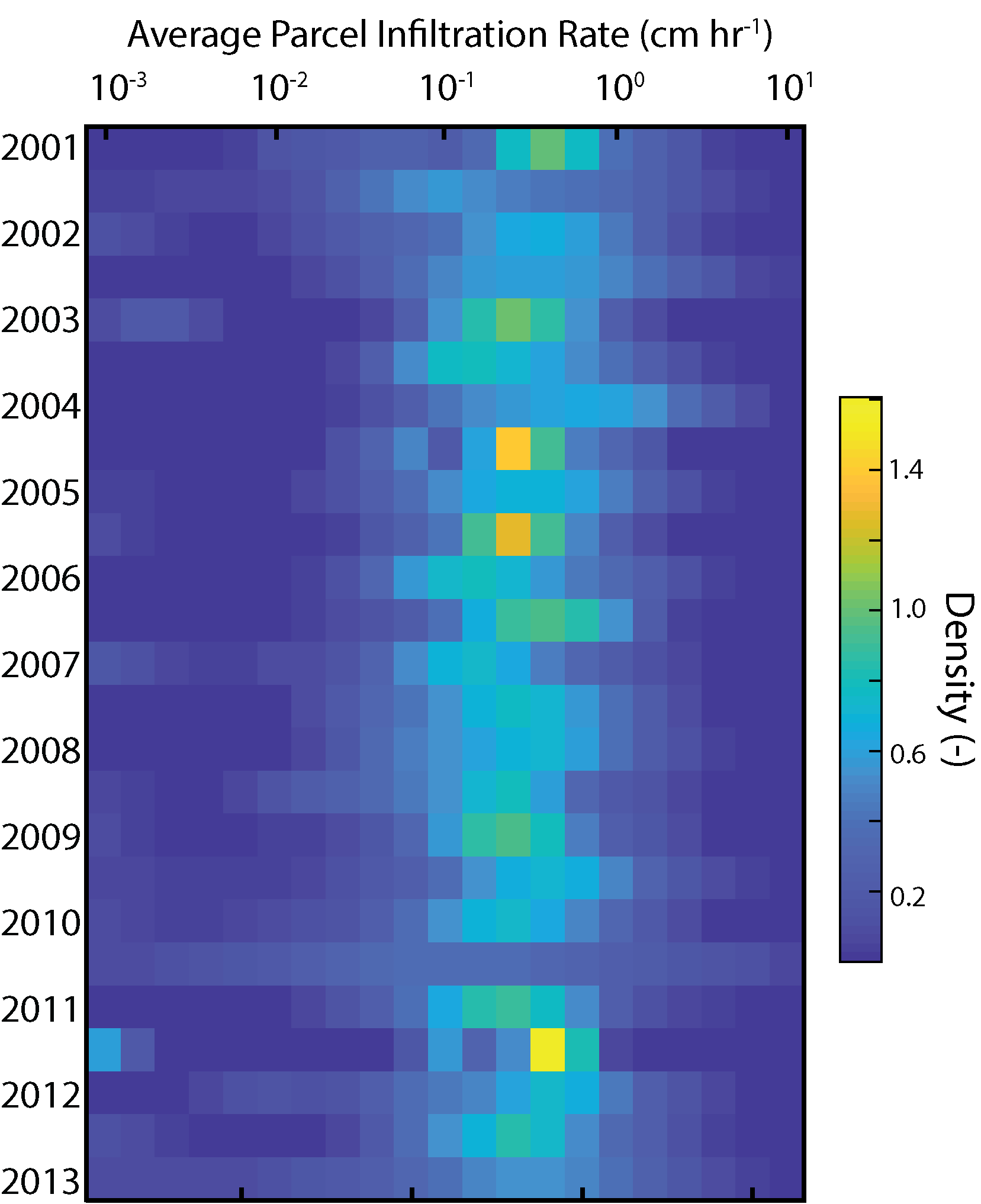


**Supplementary Figure** **2:** Probability density of average parcel infiltration rates as a function of demolition date.

**
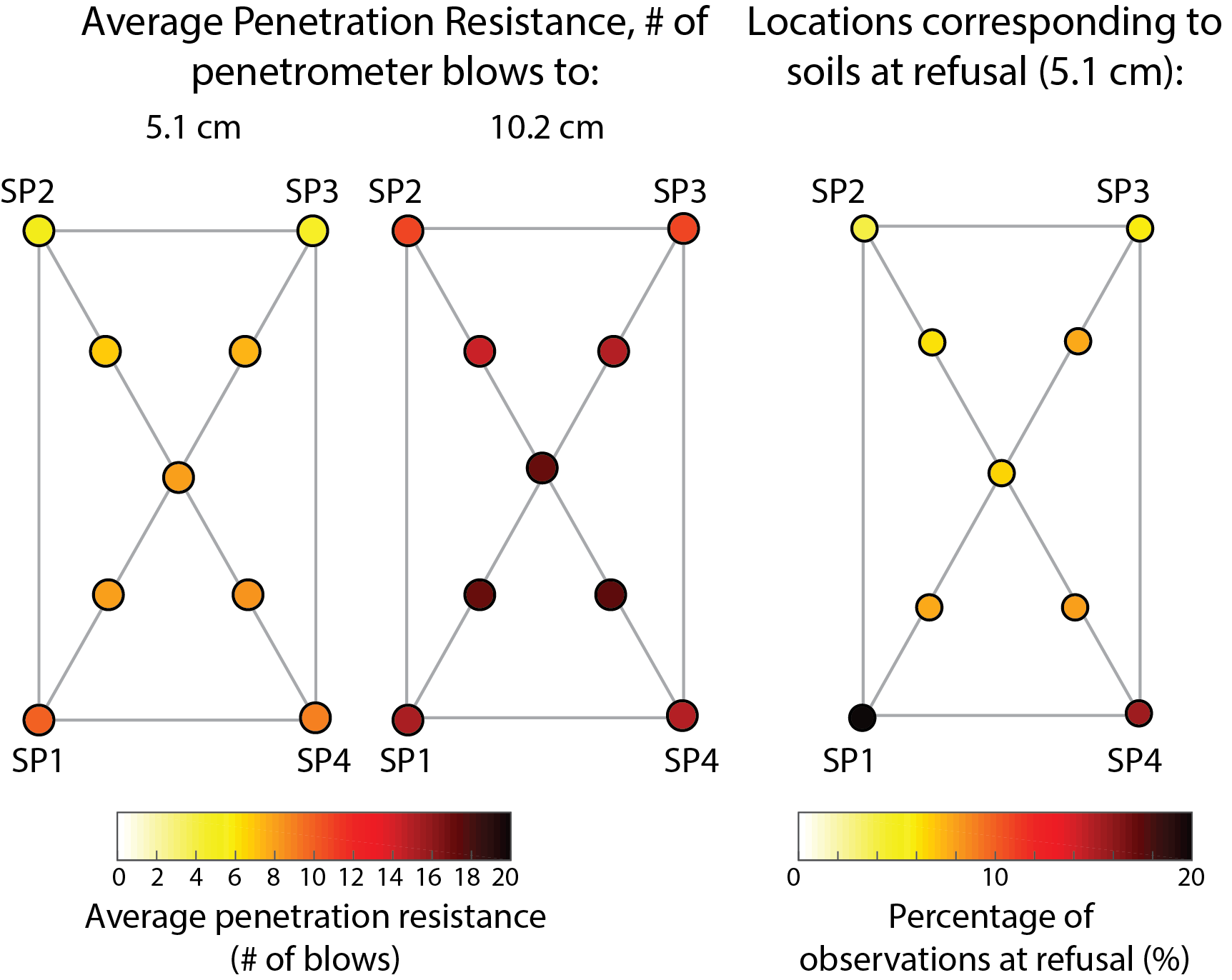
**

**Supplementary Figure** **3:** Average penetrometer observations for a typical parcel assessed by setting refusal data at 30 blows (versus 99 blows; Figure 3) for number of blows to (a) 5.1 cm and (b) 10.2 cm and (c) percentage of observations at refusal for 5.1 cm. These comparisons indicate that areas at refusal and with high compaction (e.g., more blows to compact soil) are, as shown in Figure 3, concentrated to the front of each lot. Patterns conform to those in Figure 3 (with artefacts and compact soils observed to the front of each lot), but show that treatment of refusal has potential to impact interpretation of averaged observations.


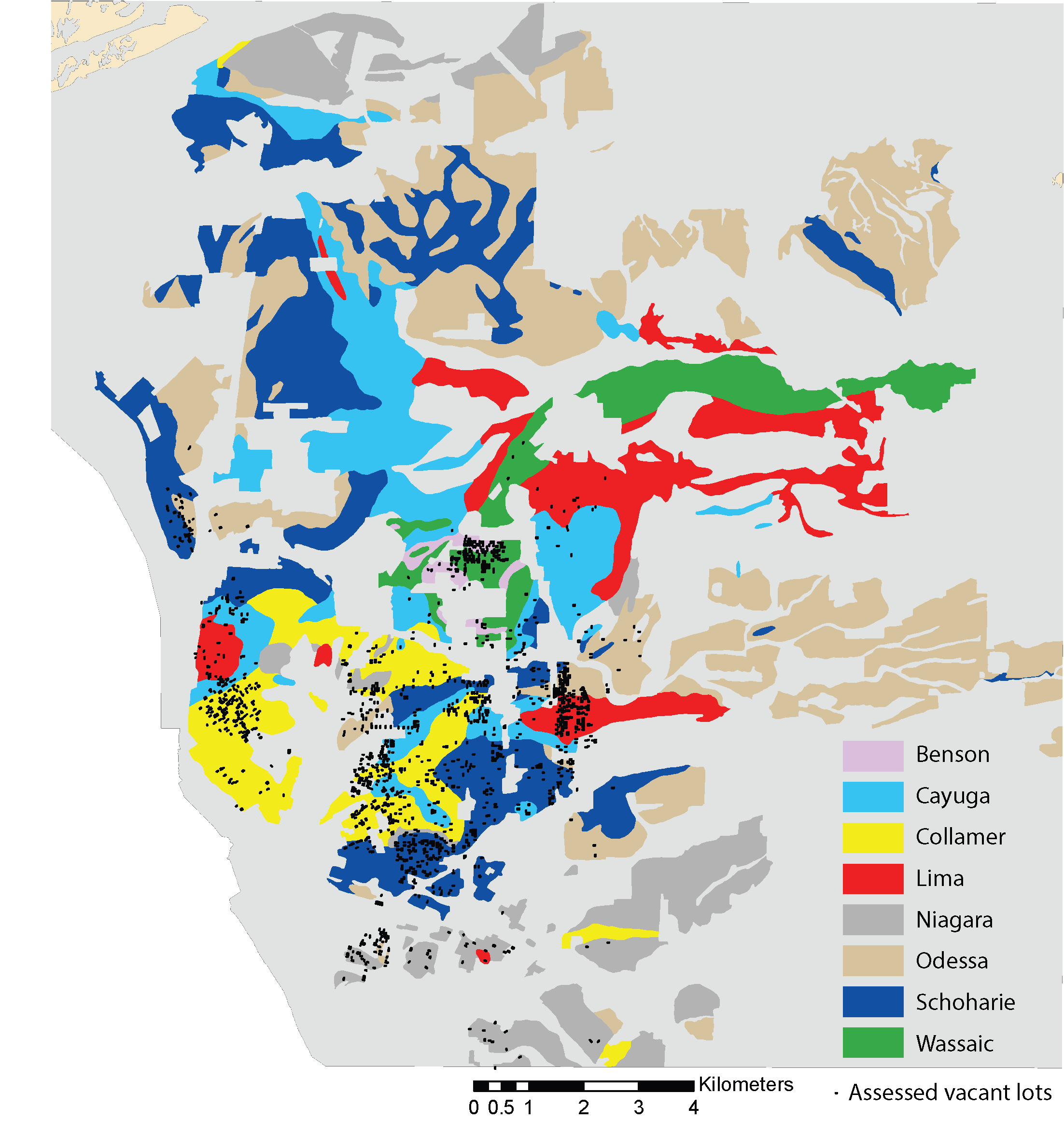
 **Supplementary Figure** **4:** Distribution of urban soil units (Supplementary Table 6) overlain by locations of vacant lots.


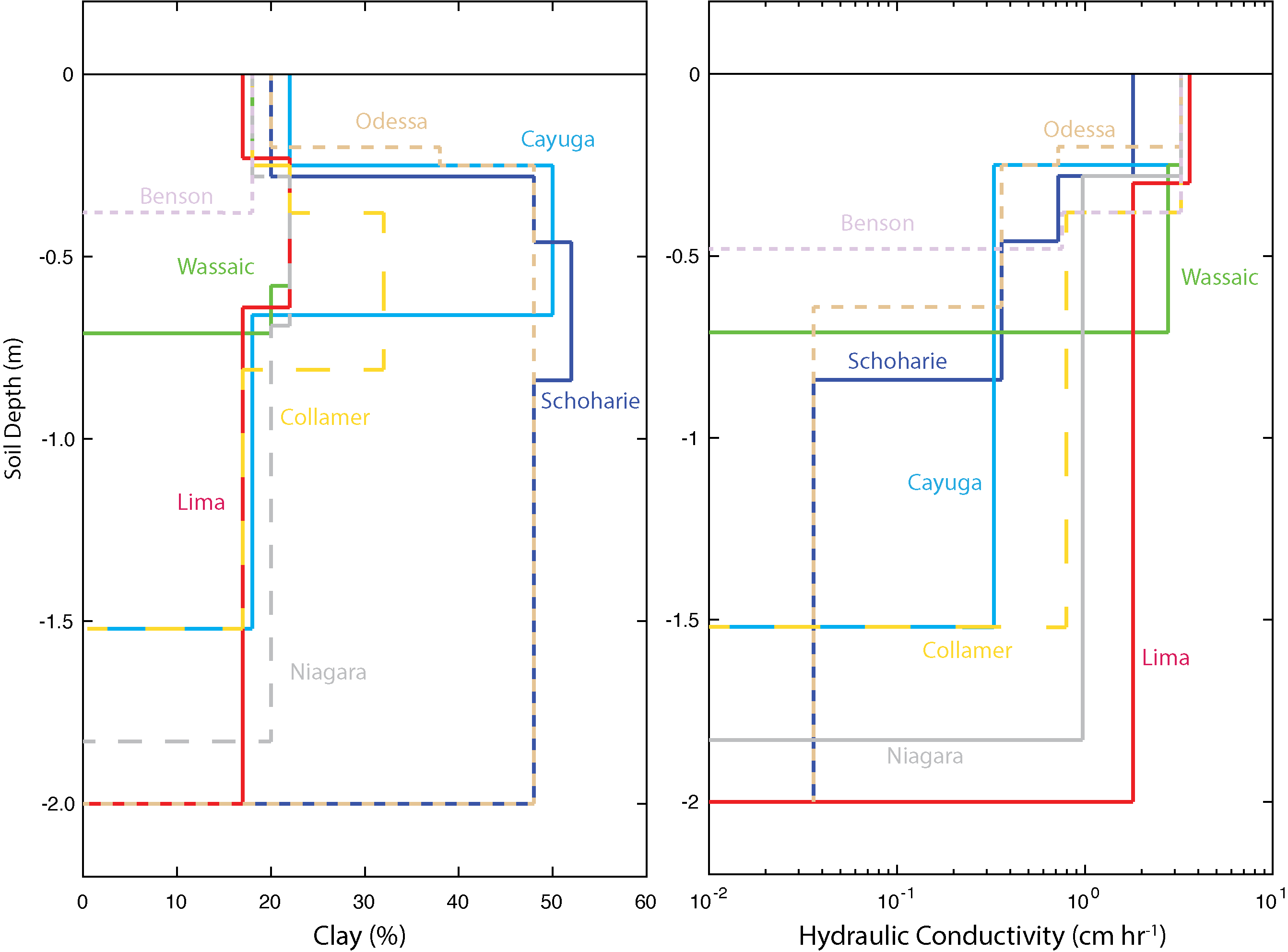


**Supplementary Figure** **5:** Variations in clay context and hydraulic conductivity with depth for reference soils (Supplementary Table 6).


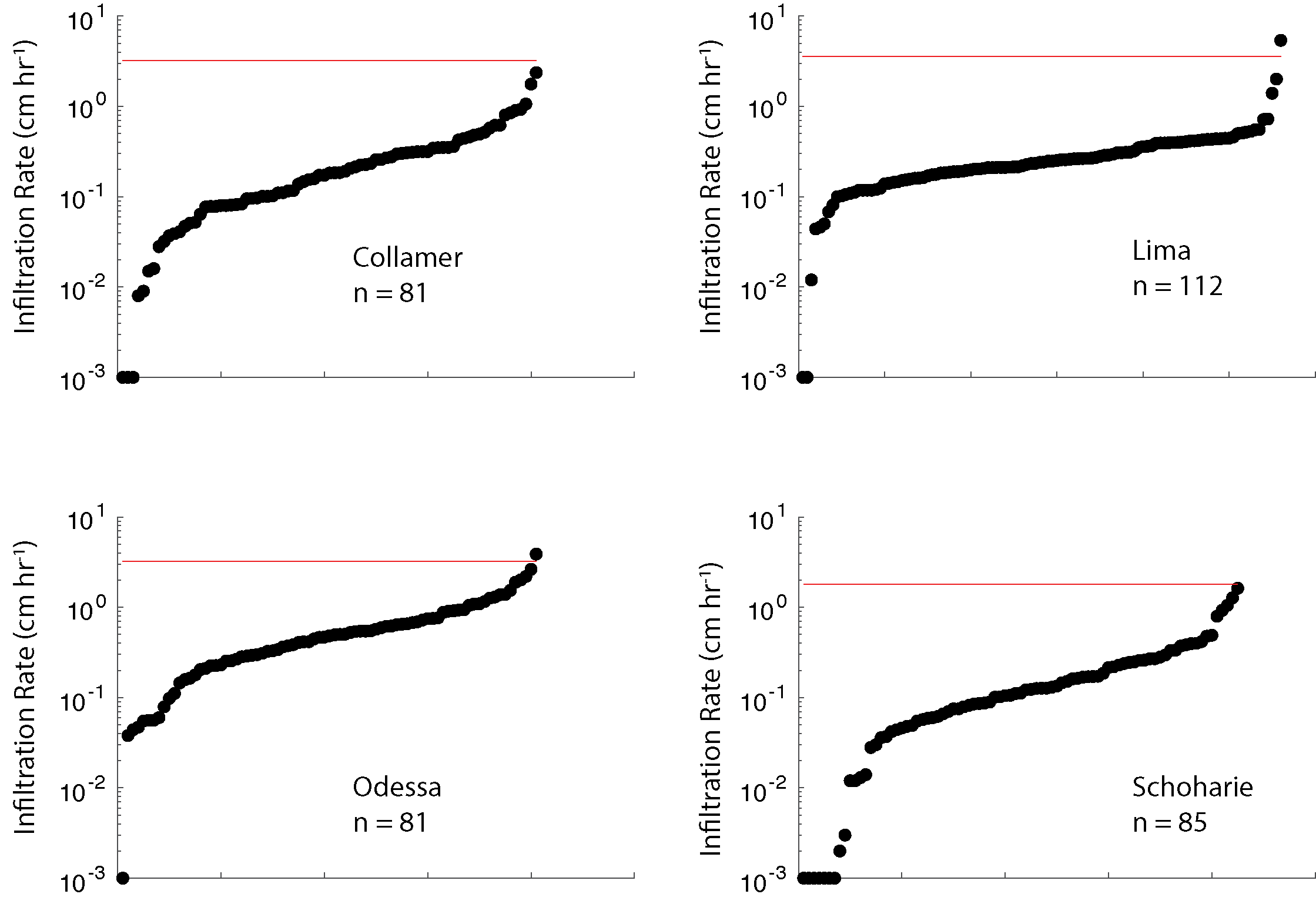


**Supplementary Figure** **6:** Reference soil hydraulic conductivity (red line) compared with corresponding urban soil infiltration rates for four select map units.


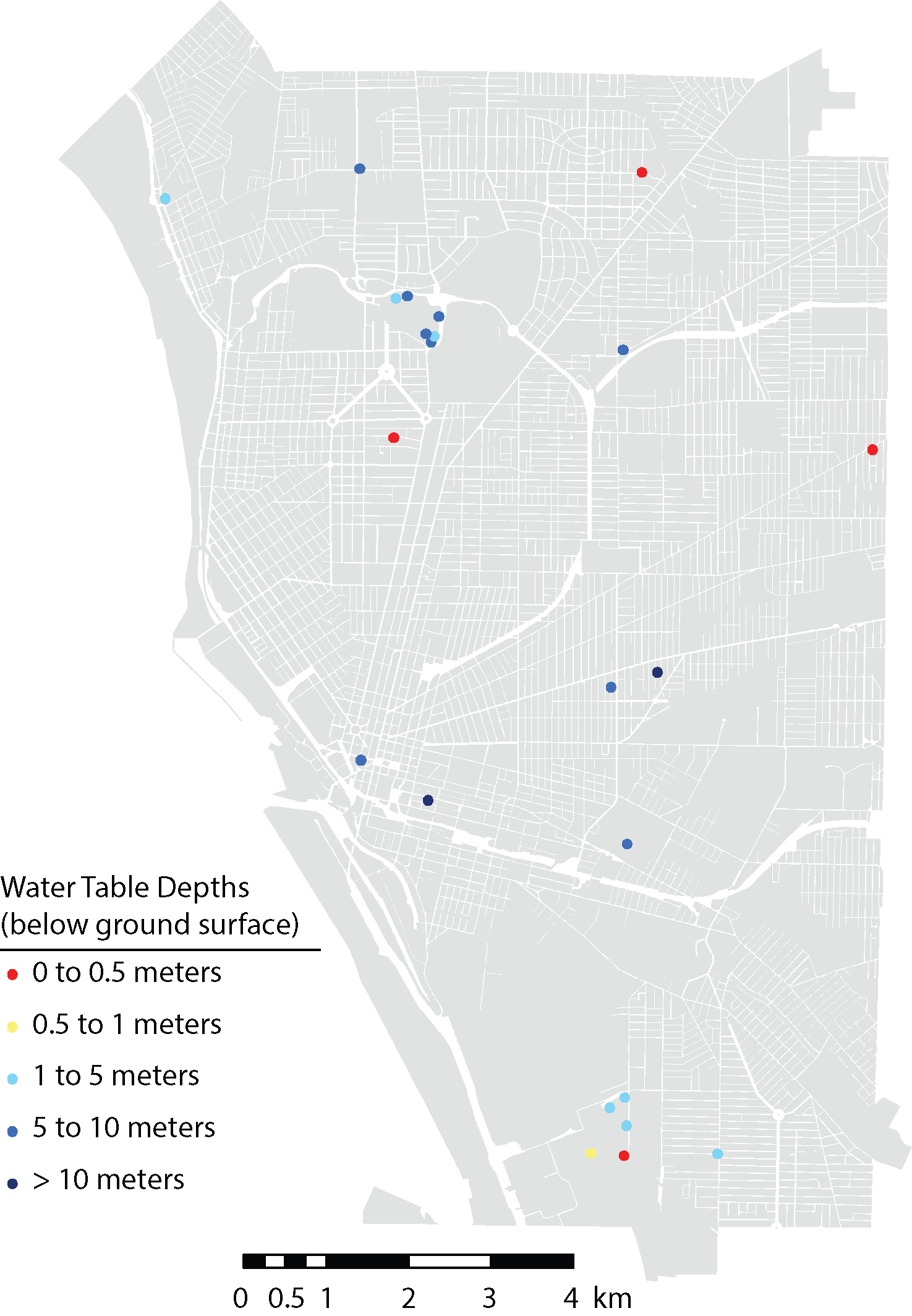


**Supplementary Figure 7:** Instantaneous groundwater levels as measured by the United States Geological Survey across Buffalo.


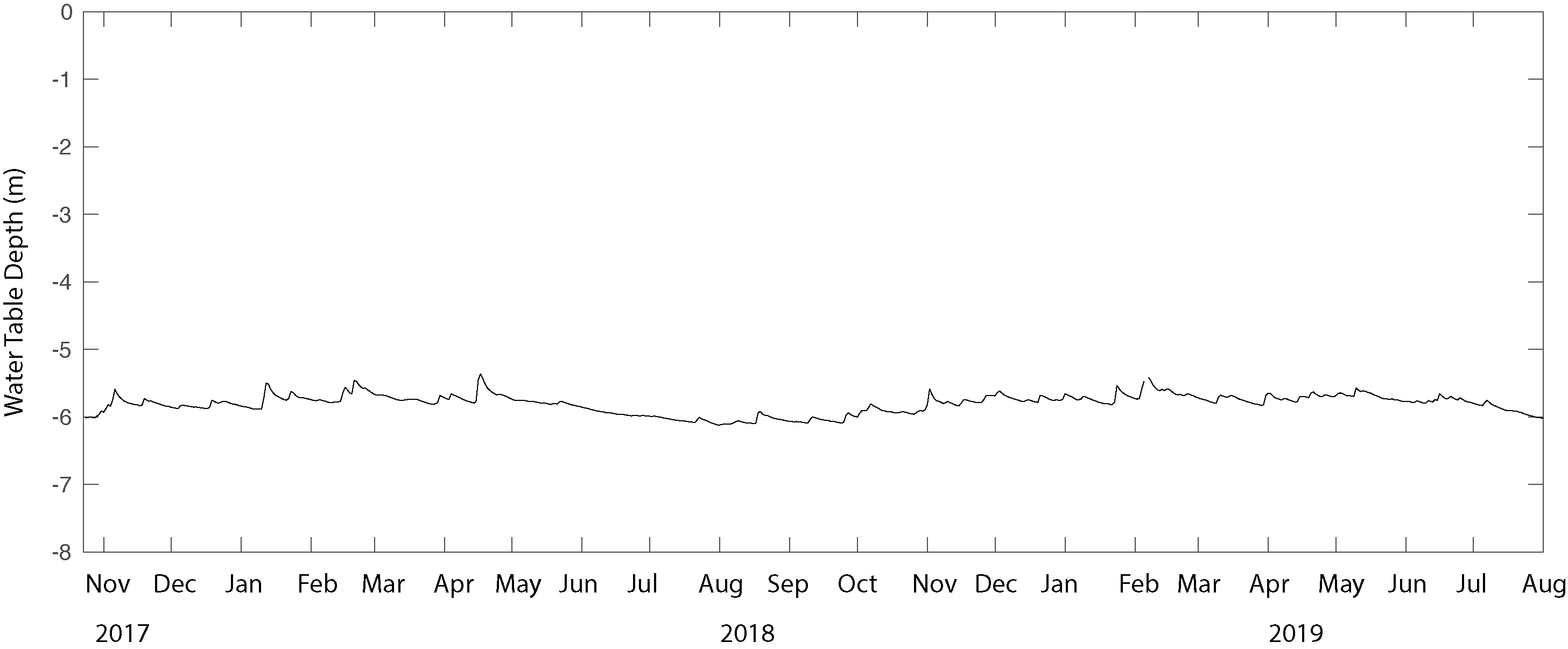


**Supplementary Figure** **8:** Timeseries of water table depth from USGS groundwater well (#430006078464101; Local number: E-1929) located on SUNY Buffalo campus.


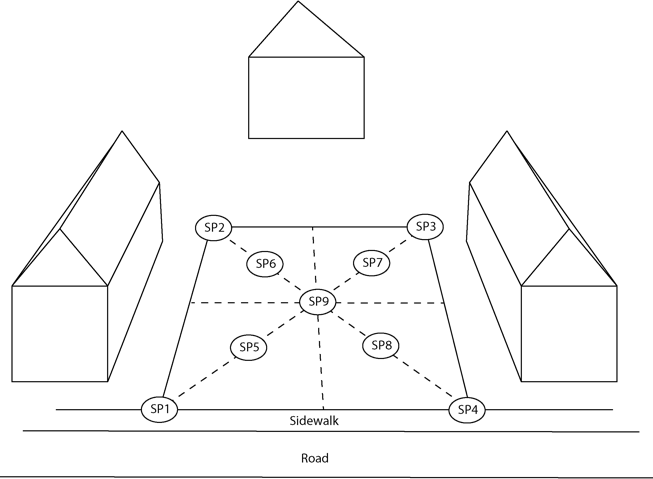


**Supplementary Figure 9:** Site layout protocol used to standardize locations of observations across each lot within the Urban Vacant Land Assessment Protocol^2^. Measurements were made at select station points (SP; 1 through 9) referenced in the figure at lot edges, diagonals, and center.


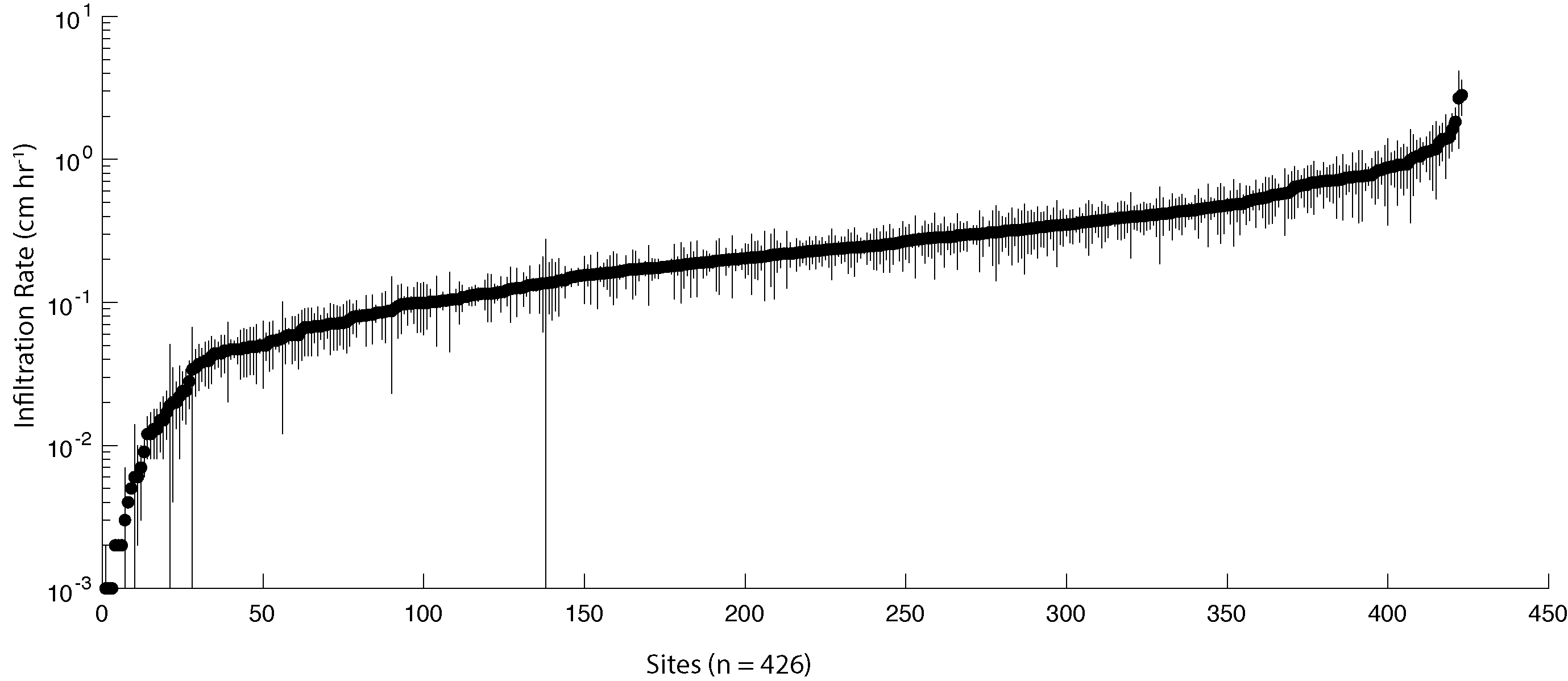


**Supplementary Figure 10:** Mean (circles) and 99% confidence intervals (lines) of infiltration rates from propagating measurement uncertainty (± 1 mL) through timeseries of infiltrometer water levels (n = 426).


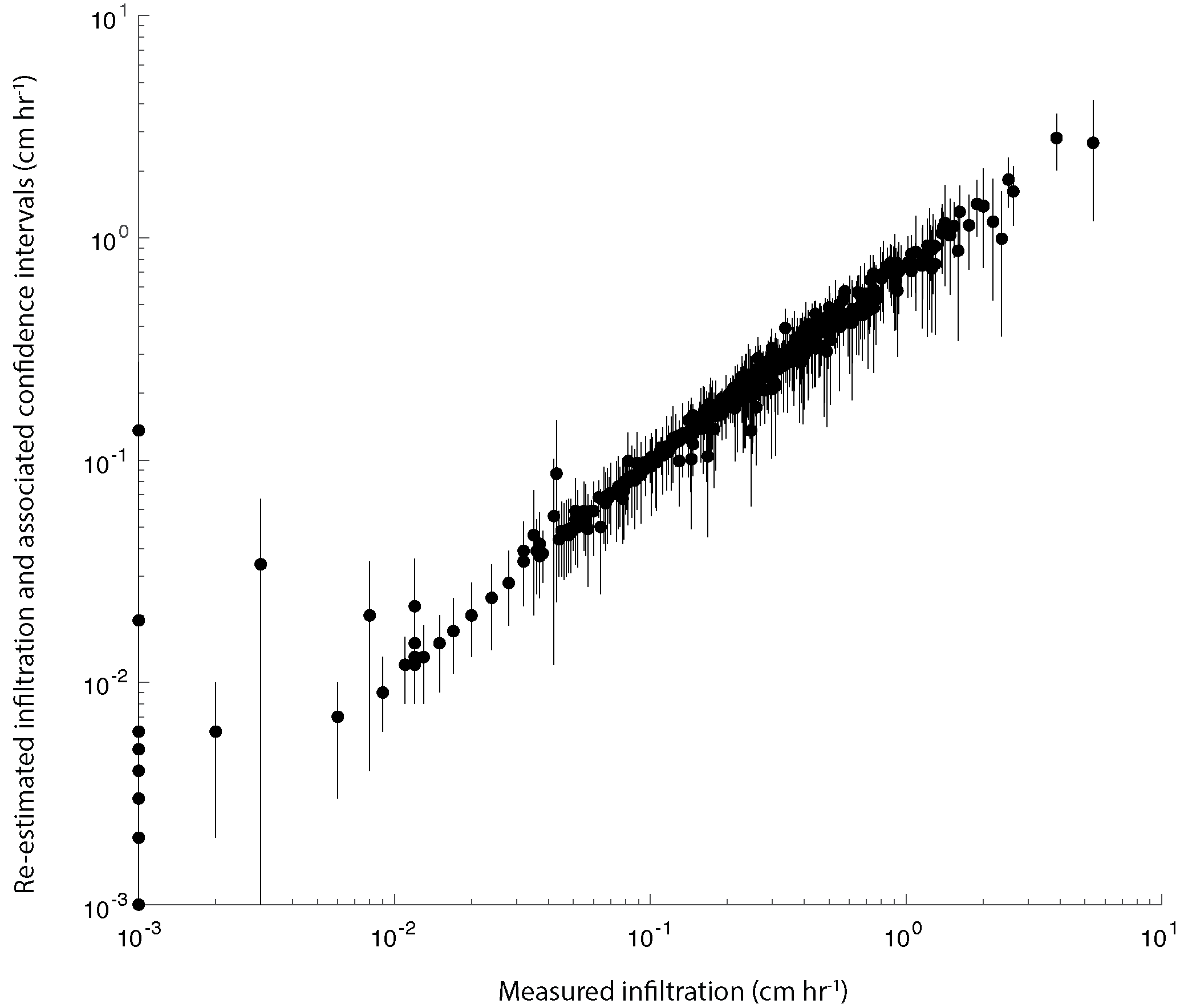


**Supplementary Figure 11:** Mean infiltration rate and 99% confidence intervals (y-axis) as compared to measured infiltration rate (n = 426).


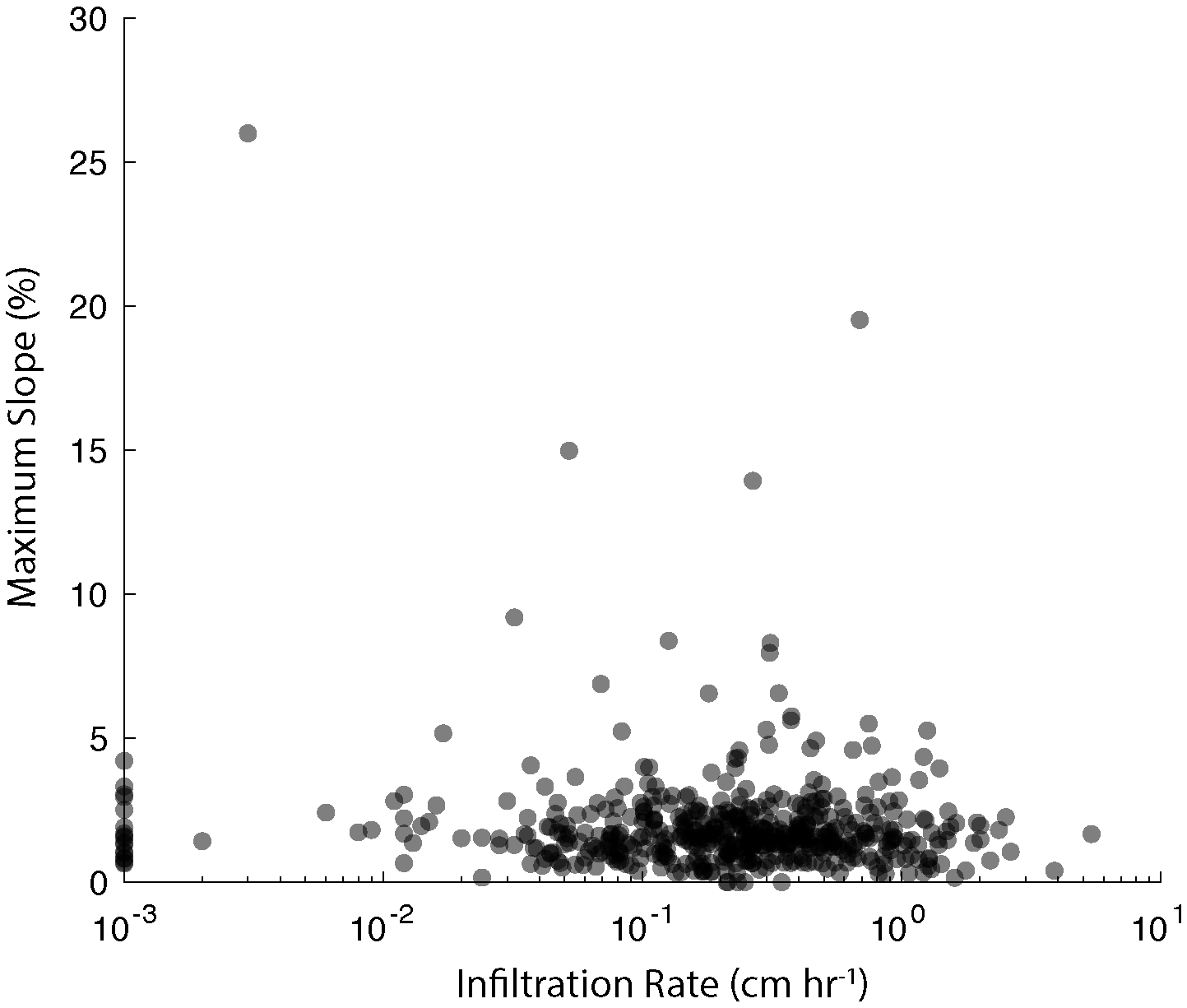


**Supplementary Figure** **12:** Infiltration rate versus maximum parcel slope.


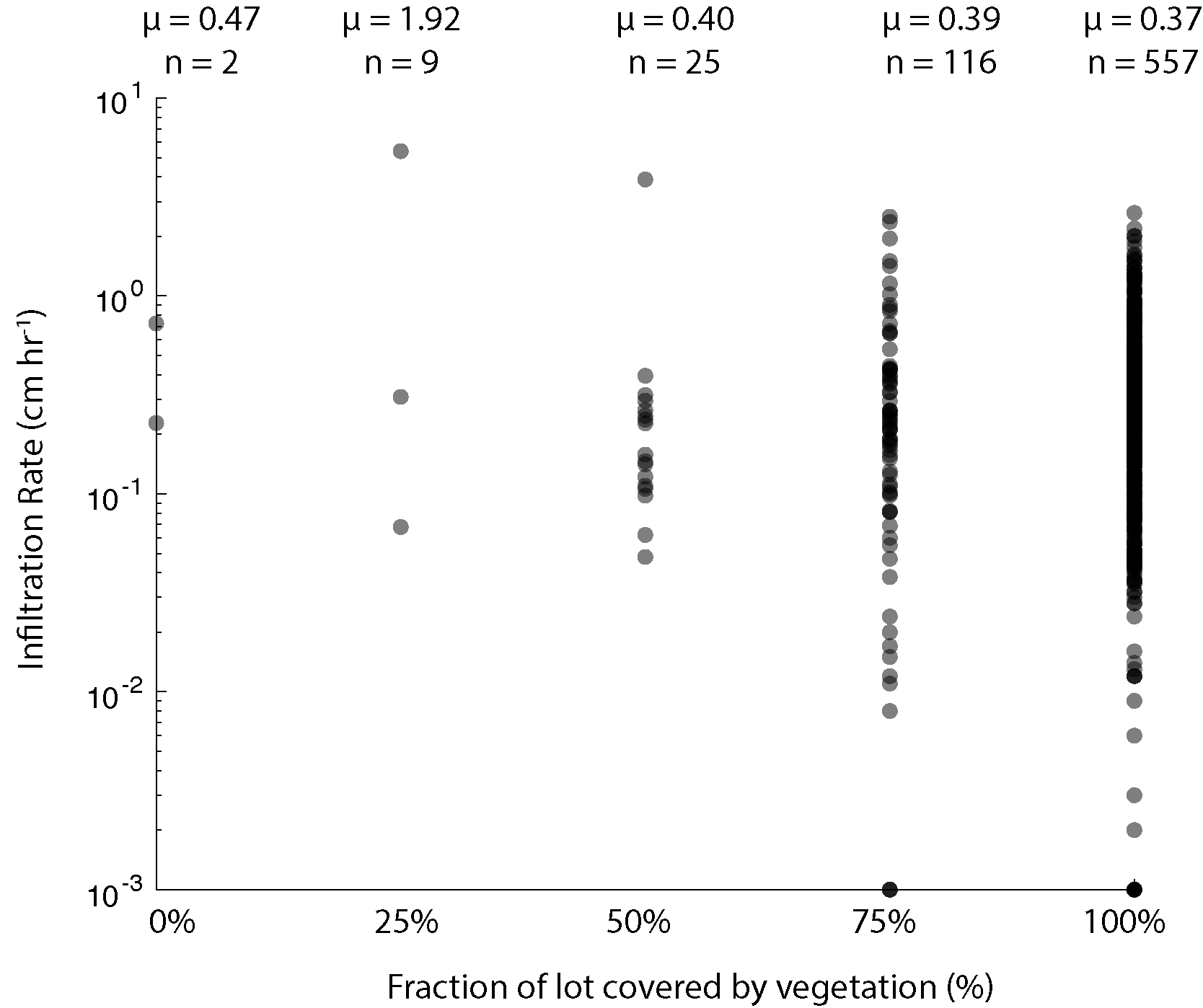


**Supplementary Figure** **13:** Infiltration rate versus fraction of lot covered by vegetation (%).

**Supplementary Tables:**

**Supplementary Table 1:** Number of parcels per unique sewershed for sites with two infiltration measurements (520 parcels) and sites with quantitative information on land cover, compaction, and topography (718 parcels).

| Sewershed | Parcels per sewershed  (520 parcels) | Parcels per sewershed  (718 parcels) |
| --- | --- | --- |
| 3 | 7 | 7 |
| 4 | 4 | 5 |
| 6 | 9 | 9 |
| 12 | 12 | 18 |
| 13 | 0 | 1 |
| 15 | 10 | 11 |
| 17 | 110 | 134 |
| 25 | 5 | 5 |
| 26 | 83 | 172 |
| 27 | 0 | 10 |
| 33 | 76 | 96 |
| 53 | 130 | 156 |
| 55 | 67 | 82 |
| 59 | 1 | 2 |
| 64 | 9 | 10 |

**Supplementary Table 2:** Spatial autocorrelation of average infiltration rates, for all sites and split out for five sewersheds.

|  | All | CSO17 | CSO26 | CSO33 | CSO53 | CSO55 |
| --- | --- | --- | --- | --- | --- | --- |
|  | n = 520 | n = 108 | n = 82 | n = 76 | n = 130 | n = 67 |
| Moran’s I | 0.110 | 0.042 | 0.231 | 0.014 | 0.174 | 0.067 |
| p-value | <0.001 | 0.12 | <0.001 | 0.54 | <0.001 | 0.12 |
| Z-score | 8.60 | 1.55 | 3.62 | 0.62 | 8.81 | 1.55 |

**Supplementary Table 3:** Spatial autocorrelation of log-transformed average infiltration rates, for all sites and split out for five sewersheds.

|  | All | CSO17 | CSO26 | CSO33 | CSO53 | CSO55 |
| --- | --- | --- | --- | --- | --- | --- |
|  | n = 520 | n = 108 | n = 82 | n =76 | n = 130 | n = 67 |
| Moran’s I | 0.153 | 0.126 | 0.124 | 0.001 | 0.156 | 0.038 |
| p-value | <0.001 | <0.001 | 0.06 | 0.81 | <0.001 | 0.32 |
| Z-score | 11.60 | 3.86 | 1.91 | 0.24 | 7.96 | 1.00 |

**Supplementary Table 4.** Linear (r^2^) and nonlinear ($\rho$) relationships between infiltration rate and log-transformed infiltration rate and demolition age (expressed in terms of the number of months since January 2001).

|  | Infiltration Rate | Log_10_(Infiltration Rate) |
| --- | --- | --- |
| Spearman’s Rank, $\rho$ | -0.037 | -0.037 |
| Coefficient of determination (r^2^) | < 0.001 | 0.002 |

**Supplementary Table 5:** The percentage of lots (ca. 718 properties, 100% of full assessment parcel dataset) with between 0 and 100% vegetated cover.

|  | Percentage of Lot | | | | |
| --- | --- | --- | --- | --- | --- |
|  | 0% | 25% | 50% | 75% | 100% |
| Vegetated | 2 | 9 | 25 | 116 | 557 |

**Supplementary Table 6:** Descriptive and quantitative information for reference soils and corresponding urban (“impacted”) units.

| Reference Map Unit | Urban Map Unit | **Depth Range (cm)** | **Horizon Designation** | **Percent Clay** | **Percent Sand** | **Hydraulic Conductivity (mm/hr)** |
| --- | --- | --- | --- | --- | --- | --- |
| Benson very channery loam  (3 to 8% slopes) | Urban land, Benson complex  (3 to 6% slopes) | 0 - 15 | H1 | 18 | 42.1 | 32.4 |
|  |  | 15 - 38 | H2 | 18 | 43.2 | 32.4 |
|  |  | 38 - 48 | H3 | - | - | 7.56 |
| Cayuga silt loam  (3 to 8 % slopes) | Urban land,  Cayuga complex | 0 - 25 | H1 | 22 | 24 | 32.4 |
|  |  | 25 - 66 | H2 | 50 | 5.3 | 3.276 |
|  |  | 66 - 152 | H3 | 18 | 43.2 | 3.276 |
| Collamer silt loam  (3 to 8 percent slopes) | Urban land, Collamer complex (1 to 6 % slopes) | 0 - 25 | H1 | 18 | 11.3 | 32.4 |
|  |  | 25 - 38 | H2 | 22 | 11.2 | 32.4 |
|  |  | 38 - 81 | H3 | 32 | 6.3 | 7.956 |
|  |  | 81 - 152 | H4 | 17 | 40 | 7.956 |
| Lima loam  (3 to 8 % slopes) | Urban land,  Lima complex  (1 to 6 % slopes) | 0 - 23 | Ap | 17 | 41 | 36 |
|  |  | 23 - 30 | Bt/E | 22 | 37 | 36 |
|  |  | 30 - 41 | Bt1 | 22 | 37 | 18 |
|  |  | 41 - 64 | Bt2 | 22 | 37 | 18 |
|  |  | 64 - 200 | C | 17 | 41 | 18 |
| Niagara silt loam  (0 to 3% slopes) | Urban land,  Niagara complex | 0 - 28 | H1 | 18 | 11.4 | 32.4 |
|  |  | 28 - 69 | H2 | 22 | 11.2 | 9.72 |
|  |  | 69 - 183 | H3 | 20 | 11.4 | 9.72 |
| Odessa silt loam  (0 to 3 % slopes) | Urban land,  Odessa complex | 0 - 20 | Ap | 20 | 15 | 32.4 |
|  |  | 20 - 25 | Bt/E | 38 | 11 | 7.2 |
|  |  | 25 - 38 | Bt1 | 48 | 6 | 3.6 |
|  |  | 38 - 64 | Bt2 | 48 | 6 | 3.6 |
|  |  | 64 - 200 | C | 48 | 6 | 0.36 |
| Schoharie silt loam  (0 to 3% slopes) | Urban land, Schoharie complex  (0 to 3% slopes) | 0 - 20 | Ap | 20 | 15 | 18 |
|  |  | 20 - 28 | E | 20 | 15 | 18 |
|  |  | 28 - 46 | Bt/E | 48 | 6 | 7.2 |
|  |  | 46 - 84 | Bt | 52 | 13 | 3.6 |
|  |  | 84 - 132 | C1 | 48 | 6 | 0.36 |
|  |  | 132 - 200 | C2 | 48 | 6 | 0.36 |
| Wassaic silt loam  (0 to 3% slopes) | Urban land, Wassaic complex | 0 - 25 | H1 | 18 | 27.1 | 32.4 |
|  |  | 25 - 58 | H2 | 22 | 26 | 27.72 |
|  |  | 58 - 71 | C | 20 | 42.1 | 27.72 |
|  |  | 71 - 81 | R | - | - | 0.018 |

**Supplementary Table 7:** Fraction of warm season precipitation (P_w_) estimated to be infiltrated across 1000 resampled datasets of infiltration rate per year, comparing post-demolition and redevelopment assessments to pre-demolition land cover.

| Year | Total P_w_ (cm) | Fraction of P_w_ Infiltrated Post-Demolition, Compared to Pre-Demolition | | | Fraction of P_w_ Infiltrated under Redevelopment, Compared to Pre-Demolition | | |
| --- | --- | --- | --- | --- | --- | --- | --- |
|  |  |  |  |  |  |  |  |
|  |  |  |  |  |  |  |  |
|  |  | Mean | Minimum | Maximum | Mean | Minimum | Maximum |
| 1997 | 49.4 | 1.52 | 1.52 | 1.54 | 1.52 | 1.51 | 1.53 |
| 1998 | 47.6 | 1.52 | 1.52 | 1.54 | 1.52 | 1.51 | 1.53 |
| 1999 | 47.8 | 1.52 | 1.52 | 1.54 | 1.52 | 1.51 | 1.53 |
| 2000 | 66.1 | 1.52 | 1.52 | 1.54 | 1.52 | 1.51 | 1.53 |
| 2001 | 44.2 | 1.52 | 1.52 | 1.54 | 1.52 | 1.51 | 1.53 |
| 2002 | 54.8 | 1.52 | 1.52 | 1.54 | 1.52 | 1.51 | 1.53 |
| 2003 | 54.7 | 1.52 | 1.52 | 1.54 | 1.52 | 1.51 | 1.53 |
| 2004 | 67.1 | 1.52 | 1.52 | 1.54 | 1.52 | 1.51 | 1.53 |
| 2005 | 58.8 | 1.52 | 1.51 | 1.54 | 1.52 | 1.51 | 1.53 |
| 2006 | 73.7 | 1.52 | 1.52 | 1.54 | 1.52 | 1.51 | 1.53 |
| 2007 | 41.5 | 1.52 | 1.52 | 1.54 | 1.52 | 1.51 | 1.53 |
| 2008 | 63.0 | 1.52 | 1.52 | 1.54 | 1.52 | 1.51 | 1.53 |
| 2009 | 70.3 | 1.52 | 1.52 | 1.54 | 1.52 | 1.51 | 1.53 |
| 2010 | 60.9 | 1.52 | 1.51 | 1.54 | 1.52 | 1.51 | 1.54 |
| 2011 | 84.1 | 1.52 | 1.52 | 1.54 | 1.52 | 1.51 | 1.53 |
| 2012 | 49.0 | 1.52 | 1.52 | 1.54 | 1.52 | 1.51 | 1.53 |
| 2013 | 79.8 | 1.52 | 1.51 | 1.54 | 1.52 | 1.51 | 1.54 |
| 2014 | 70.6 | 1.52 | 1.51 | 1.54 | 1.52 | 1.51 | 1.53 |
| 2015 | 66.2 | 1.52 | 1.52 | 1.54 | 1.52 | 1.51 | 1.53 |
| 2016 | 46.1 | 1.52 | 1.52 | 1.54 | 1.52 | 1.51 | 1.53 |
| 2017 | 78.3 | 1.52 | 1.51 | 1.54 | 1.52 | 1.51 | 1.54 |

**Supplementary Table 8:** Fraction of warm season precipitation (P_w_) estimated to be converted to runoff. We based these estimates on 1000 resampled datasets of infiltration rate, per year, comparing post-demolition and redevelopment assessments to pre-demolition land cover.

| Year | Total P_w_ (cm) | Fraction of P_w_ Estimated as Runoff Post-Demolition, Compared to Pre-Demolition | | | Fraction of P_w_ Estimated as Runoff in Redevelopment, Compared to Pre-Demolition | | |
| --- | --- | --- | --- | --- | --- | --- | --- |
|  |  |  |  |  |  |  |  |
|  |  |  |  |  |  |  |  |
|  |  | Mean | Minimum | Maximum | Mean | Minimum | Maximum |
| 1997 | 49.4 | 0.59 | 0.57 | 0.61 | 0.59 | 0.58 | 0.62 |
| 1998 | 47.6 | 0.64 | 0.62 | 0.66 | 0.64 | 0.62 | 0.66 |
| 1999 | 47.8 | 0.64 | 0.63 | 0.66 | 0.64 | 0.63 | 0.66 |
| 2000 | 66.1 | 0.62 | 0.60 | 0.64 | 0.62 | 0.60 | 0.64 |
| 2001 | 44.2 | 0.56 | 0.54 | 0.58 | 0.56 | 0.54 | 0.58 |
| 2002 | 54.8 | 0.58 | 0.56 | 0.60 | 0.59 | 0.57 | 0.61 |
| 2003 | 54.7 | 0.60 | 0.58 | 0.62 | 0.60 | 0.58 | 0.62 |
| 2004 | 67.1 | 0.66 | 0.65 | 0.68 | 0.66 | 0.65 | 0.68 |
| 2005 | 58.8 | 0.68 | 0.67 | 0.70 | 0.68 | 0.67 | 0.7 |
| 2006 | 73.7 | 0.64 | 0.63 | 0.66 | 0.64 | 0.63 | 0.66 |
| 2007 | 41.5 | 0.62 | 0.61 | 0.64 | 0.63 | 0.61 | 0.65 |
| 2008 | 63.0 | 0.66 | 0.65 | 0.68 | 0.66 | 0.65 | 0.68 |
| 2009 | 70.3 | 0.66 | 0.65 | 0.68 | 0.66 | 0.65 | 0.68 |
| 2010 | 60.9 | 0.69 | 0.68 | 0.71 | 0.69 | 0.68 | 0.71 |
| 2011 | 84.1 | 0.65 | 0.64 | 0.67 | 0.65 | 0.64 | 0.67 |
| 2012 | 49.0 | 0.59 | 0.57 | 0.61 | 0.59 | 0.57 | 0.61 |
| 2013 | 79.8 | 0.70 | 0.68 | 0.71 | 0.70 | 0.68 | 0.72 |
| 2014 | 70.6 | 0.66 | 0.65 | 0.68 | 0.66 | 0.65 | 0.68 |
| 2015 | 66.2 | 0.67 | 0.65 | 0.69 | 0.67 | 0.66 | 0.69 |
| 2016 | 46.1 | 0.65 | 0.64 | 0.67 | 0.66 | 0.64 | 0.68 |
| 2017 | 78.3 | 0.70 | 0.68 | 0.71 | 0.70 | 0.69 | 0.72 |

**Supplementary Table 9**: Infiltrated volume of water per year (10^5^ m^3^) pre-demolition, post-demolition, and post-redevelopment. Volumes are calculated per timeseries of warm-season precipitation (P_w_). Mean, minimum (min), and maximum (max) volumes were aggregated per year from 1000 resampled infiltration rates applied to ca. 2400 vacant lots.

| Year | P_w_  (cm) | Pre-Demolition (10^5^ m^3^) | | | Post-Demolition (10^5^ m^3^) | | | Redevelopment (10^5^ m^3^) | | |
| --- | --- | --- | --- | --- | --- | --- | --- | --- | --- | --- |
|  |  | Mean | Max | Min | Mean | Max | Min | Mean | Max | Min |
| 1997 | 49.4 | 1.85 | 1.91 | 1.78 | 2.81 | 2.90 | 2.72 | 2.80 | 2.89 | 2.71 |
| 1998 | 47.6 | 1.66 | 1.72 | 1.60 | 2.53 | 2.61 | 2.44 | 2.52 | 2.60 | 2.43 |
| 1999 | 47.8 | 1.66 | 1.71 | 1.59 | 2.53 | 2.61 | 2.44 | 2.52 | 2.60 | 2.43 |
| 2000 | 66.1 | 2.38 | 2.47 | 2.29 | 3.63 | 3.75 | 3.50 | 3.62 | 3.74 | 3.49 |
| 2001 | 44.2 | 1.73 | 1.78 | 1.67 | 2.63 | 2.71 | 2.55 | 2.63 | 2.70 | 2.54 |
| 2002 | 54.8 | 2.07 | 2.15 | 2.00 | 3.16 | 3.26 | 3.05 | 3.15 | 3.25 | 3.04 |
| 2003 | 54.7 | 2.03 | 2.09 | 1.96 | 3.10 | 3.18 | 2.99 | 3.09 | 3.17 | 2.98 |
| 2004 | 67.1 | 2.24 | 2.32 | 2.15 | 3.42 | 3.53 | 3.29 | 3.41 | 3.52 | 3.28 |
| 2005 | 58.8 | 1.90 | 1.97 | 1.82 | 2.90 | 3.00 | 2.79 | 2.89 | 2.99 | 2.78 |
| 2006 | 73.7 | 2.55 | 2.64 | 2.45 | 3.89 | 4.02 | 3.74 | 3.87 | 4.00 | 3.73 |
| 2007 | 41.5 | 1.48 | 1.53 | 1.42 | 2.25 | 2.32 | 2.17 | 2.24 | 2.31 | 2.17 |
| 2008 | 63.0 | 2.10 | 2.18 | 2.02 | 3.21 | 3.32 | 3.09 | 3.20 | 3.31 | 3.08 |
| 2009 | 70.3 | 2.36 | 2.44 | 2.26 | 3.59 | 3.71 | 3.46 | 3.58 | 3.70 | 3.45 |
| 2010 | 60.9 | 1.93 | 2.00 | 1.85 | 2.94 | 3.04 | 2.83 | 2.93 | 3.04 | 2.82 |
| 2011 | 84.1 | 2.86 | 2.97 | 2.75 | 4.36 | 4.52 | 4.20 | 4.35 | 4.51 | 4.18 |
| 2012 | 49.0 | 1.84 | 1.90 | 1.77 | 2.80 | 2.89 | 2.70 | 2.79 | 2.88 | 2.70 |
| 2013 | 79.8 | 2.50 | 2.60 | 2.39 | 3.81 | 3.95 | 3.65 | 3.80 | 3.94 | 3.64 |
| 2014 | 70.6 | 2.36 | 2.46 | 2.27 | 3.60 | 3.73 | 3.46 | 3.59 | 3.72 | 3.45 |
| 2015 | 66.2 | 2.19 | 2.28 | 2.10 | 3.34 | 3.46 | 3.21 | 3.33 | 3.45 | 3.20 |
| 2016 | 46.1 | 1.56 | 1.62 | 1.50 | 2.38 | 2.47 | 2.30 | 2.38 | 2.46 | 2.29 |
| 2017 | 78.3 | 2.45 | 2.55 | 2.35 | 3.74 | 3.87 | 3.59 | 3.72 | 3.86 | 3.57 |

**Supplementary Table 10:** Volume of runoff per year (10^5^ m^3^) estimated to occur for pre-demolition, post-demolition, and redevelopment states. Volumes are calculated per timeseries of warm-season precipitation (P_w_). Mean, minimum (min), and maximum (max) volumes were aggregated per year from 1000 resampled infiltration rates applied to ca. 2400 vacant lots.

| Year | P_w_  (cm) | Pre-Demolition (10^5^ m^3^) | | | Post-Demolition (10^5^ m^3^) | | | Redevelopment (10^5^ m^3^) | | |
| --- | --- | --- | --- | --- | --- | --- | --- | --- | --- | --- |
|  |  | Mean | Max | Min | Mean | Max | Min | Mean | Max | Min |
| 1997 | 49.4 | 2.37 | 2.43 | 2.30 | 1.40 | 1.50 | 1.31 | 1.41 | 1.51 | 1.32 |
| 1998 | 47.6 | 2.40 | 2.47 | 2.35 | 1.53 | 1.63 | 1.45 | 1.54 | 1.63 | 1.46 |
| 1999 | 47.8 | 2.42 | 2.48 | 2.36 | 1.55 | 1.64 | 1.47 | 1.56 | 1.65 | 1.48 |
| 2000 | 66.1 | 3.26 | 3.35 | 3.17 | 2.01 | 2.14 | 1.89 | 2.02 | 2.15 | 1.90 |
| 2001 | 44.2 | 2.05 | 2.11 | 1.99 | 1.14 | 1.23 | 1.06 | 1.15 | 1.24 | 1.07 |
| 2002 | 54.8 | 2.60 | 2.68 | 2.53 | 1.51 | 1.63 | 1.41 | 1.52 | 1.64 | 1.42 |
| 2003 | 54.7 | 2.64 | 2.71 | 2.57 | 1.57 | 1.68 | 1.48 | 1.58 | 1.69 | 1.49 |
| 2004 | 67.1 | 3.48 | 3.57 | 3.40 | 2.31 | 2.44 | 2.19 | 2.32 | 2.45 | 2.20 |
| 2005 | 58.8 | 3.12 | 3.20 | 3.05 | 2.12 | 2.23 | 2.02 | 2.13 | 2.24 | 2.03 |
| 2006 | 73.7 | 3.74 | 3.84 | 3.65 | 2.40 | 2.55 | 2.27 | 2.41 | 2.56 | 2.28 |
| 2007 | 41.5 | 2.06 | 2.11 | 2.01 | 1.28 | 1.36 | 1.22 | 1.29 | 1.37 | 1.22 |
| 2008 | 63.0 | 3.27 | 3.35 | 3.19 | 2.16 | 2.29 | 2.06 | 2.17 | 2.30 | 2.07 |
| 2009 | 70.3 | 3.65 | 3.74 | 3.56 | 2.41 | 2.54 | 2.29 | 2.42 | 2.56 | 2.30 |
| 2010 | 60.9 | 3.26 | 3.34 | 3.19 | 2.25 | 2.37 | 2.15 | 2.26 | 2.38 | 2.16 |
| 2011 | 84.1 | 4.31 | 4.43 | 4.20 | 2.81 | 2.98 | 2.66 | 2.82 | 2.99 | 2.67 |
| 2012 | 49.0 | 2.34 | 2.41 | 2.28 | 1.38 | 1.47 | 1.29 | 1.39 | 1.48 | 1.30 |
| 2013 | 79.8 | 4.32 | 4.42 | 4.21 | 3.00 | 3.16 | 2.86 | 3.02 | 3.17 | 2.87 |
| 2014 | 70.6 | 3.66 | 3.75 | 3.57 | 2.42 | 2.56 | 2.29 | 2.43 | 2.57 | 2.30 |
| 2015 | 66.2 | 3.46 | 3.55 | 3.37 | 2.31 | 2.44 | 2.19 | 2.32 | 2.45 | 2.20 |
| 2016 | 46.1 | 2.37 | 2.43 | 2.31 | 1.55 | 1.64 | 1.47 | 1.56 | 1.65 | 1.48 |
| 2017 | 78.3 | 4.23 | 4.34 | 4.14 | 2.95 | 3.10 | 2.81 | 2.96 | 3.11 | 2.82 |

**Supplementary Table 11:** Former land use indicated by property class description of Buffalo vacant lots^1^ (n = 16,119).

|  | All Properties  *n = 16,119* | | | |
| --- | --- | --- | --- | --- |
| Land Use | Total (#) | Area (km^2^) | Total (%) | Area (%) |
| Residential Vacant Land (311, 312) | 13,683 | 5.18 | 79.8% | 34.4% |
| Commercial Vacant Land (330, 331) | 2,947 | 4.85 | 17.2% | 32.2% |
| Industrial Vacant Land (340, 341) | 477 | 4.11 | 2.8% | 27.3% |
| Urban Renewal or Slum Clearance (350) | 33 | 0.93 | 0.2% | 6.2% |
| Public Utility Vacant Land (380) | 3 | 0 | < 0.1% | < 0.1% |

**Supplementary Table 12:** Number of and percentage of parcels with a given set of measurements (IN = infiltration tests, VEG = vegetation cover, IC = impervious cover, ELE = elevation, PE = penetrometer observations, LC = land cover).

| Number of Parcels | Percentage of Parcels | Criteria |
| --- | --- | --- |
| 500 | 69.6 | Sites with all measurements (IN, VEG, IC, ELE, PE, LC) |
| 520 | 72.4 | Sites with two infiltration tests (IN) |
| 709 | 98.7 | Sites with percentages of vegetation and impervious cover (VEG, IC) |
| 713 | 99.3 | Sites with elevation (ELE) |
| 715 | 99.6 | Sites with penetrometer observations (PE) at four or more station points |
| 709 | 97.8 | Sites with land cover (LC) at nine station points |

**Supplementary Table 13.** Percentage of different soil textural classes across vacant parcels within (IN) and outside (OUT) the building footprint. The most frequent soil textural classes were sandy or silty clay loams, though we note the diverse range of soil textures identified across vacant parcels, likely owing to the diversity of soil backfill materials and their respective sources.

|  | IN | OUT | IN | OUT |
| --- | --- | --- | --- | --- |
| Clay | 26 | 19 | 6% | 4% |
| Clay Loam | 61 | 58 | 14% | 13% |
| Loam | 13 | 11 | 3% | 2% |
| Loamy Sand | 13 | 9 | 3% | 2% |
| Sand | 4 | 2 | 1% | 0% |
| Sandy Clay | 30 | 29 | 7% | 6% |
| Sandy Clay Loam | 67 | 98 | 15% | 22% |
| Sandy Loam | 42 | 60 | 9% | 13% |
| Silt Loam | 26 | 29 | 6% | 6% |
| Silty Clay | 77 | 77 | 17% | 17% |
| Silty Clay Loam | 90 | 57 | 20% | 13% |
| Total | 449 | 449 | - | - |

**Supplementary Table 14:** Total warm-season precipitation (cm) estimated from NEWA^3^ (hourly dataset) and National Climatic Data Center^4^ (NCDC; daily dataset), as well as the absolute difference between the two. This table displays an independent verification of hourly NEWA values as compared to NCDC daily precipitation estimates obtained for the Buffalo Niagara International Airport^4^. With the exception of observations from 1997, there is strong agreement between these two datasets. In nearly all cases, NEWA represents an underestimate with respect to NCDC data, suggesting our calculations may also underestimate rainfall detention capacity in wet years with frequent rainfall, when infiltration excess overland flow dominates.

|  | NEWA  (cm) | NCDC  (cm) | Absolute Difference  (cm) |
| --- | --- | --- | --- |
| 1997 | 49.4 | 56.4 | 7.0 |
| 1998 | 47.6 | 50.3 | 2.7 |
| 1999 | 47.8 | 48.9 | 1.1 |
| 2000 | 66.1 | 66.3 | 0.2 |
| 2001 | 44.2 | 44.6 | 0.4 |
| 2002 | 54.8 | 55.5 | 0.7 |
| 2003 | 54.7 | 54.9 | 0.2 |
| 2004 | 67.1 | 67.6 | 0.6 |
| 2005 | 58.8 | 60.0 | 1.2 |
| 2006 | 73.7 | 78.3 | 4.6 |
| 2007 | 41.5 | 41.6 | 0.1 |
| 2008 | 63.0 | 65.3 | 2.3 |
| 2009 | 70.3 | 71.3 | 1.0 |
| 2010 | 60.9 | 60.9 | 0.0 |
| 2011 | 84.1 | 84.3 | 0.2 |
| 2012 | 49.0 | 51.0 | 2.1 |
| 2013 | 79.8 | 80.1 | 0.3 |
| 2014 | 70.6 | 72.0 | 1.4 |
| 2015 | 66.2 | 66.4 | 0.2 |
| 2016 | 46.1 | 46.6 | 0.5 |
| 2017 | 78.3 | 78.5 | 0.2 |

**Supplementary Notes:**

**Supplementary Note 1:**

To determine how reference soils, defined as soil complexes that have not been impacted by humans, compared with urban soils, we extracted information for pre-urbanization, reference soils from the Soil Survey Geographic Database (SSURGO), which is the best-known source for this type of data. For each of eight commonly occurring urban soil units, we identified the official map unit that is specified that the urban complex, and looked for the best matching unit to use as a reference^9^. For each reference unit, we extracted National Cooperative Soil Survey (NCSS) soil data from the Series Extent Explorer (<https://casoilresource.lawr.ucdavis.edu/see/>) (Supplementary Table 6), and their areal extents are shown in Supplementary Figure 4.

We analyzed two pieces of information for all reference soils: the percentage of clay by depth, and saturated hydraulic conductivity by depth. From reference soils data (Supplementary Table 6), we observed that of the eight most common urban soil complexes, corresponding reference soils infrequently displayed a high percentage of clay between 0.25 and 0.75 meters below the surface (Supplementary Figure 5). This was true for the Cayuga (50% clay), Schoharie (48 – 52% clay), and Odessa (48% clay) complexes. This data suggests that restrictive layers as clay lenses could exist in some urban soils, potentially restricting vertical movement of water, and limiting storage. However, the vast majority of reference soils displayed soil texture with less clay content (<25%). Yet, given that reference soils were disturbed through development and demolition-backfill phases of urbanization, these clay-rich layers were removed or otherwise mixed and displaced from their native condition. This assumption would also be supported by recently published work across urban areas that has found B horizons, the most common location for clay lenses in these areas, are often absent in urbanized soils^5^.

As can be seen in Supplementary Figure 5, saturated hydraulic conductivity in reference soils decreases with depth. To assess how urbanization may impact infiltration rates, we compared reference unit hydraulic conductivities in the horizon closest to the surface with vacant lot infiltration rates from the corresponding urban soil unit (Supplementary Figure 6). For all but one vacant lot site, reference hydraulic conductivities were typically orders of magnitude higher than observed infiltration rates.

We undertook this analysis to compare vacant lot soil characteristics to the best-known (USDA) reference information, so that we can then contextualize how urbanization has altered local Buffalo NY soils. However, unless the county soil survey has been recently updated (e.g., Wayne County MI, USA) to account for new and modified soil series brought about by urbanization, the use of SSURGO soils data to represent urban areas is not recommended. It is important to note that SSURGO does not include the effects of urbanization on soil complexes, hence our use of SSURGO information as a reference dataset.

**Supplementary Note 2:**

In our analysis, we apply a simple conceptual model that partitions rainfall between infiltration and runoff across vacant lots as a function of parcel area, infiltration rate, precipitation rate, and impervious surface cover. However, we also expect that this partitioning will be a function of and complex interactions amongst antecedent moisture (and therefore soil moisture and water table height), topography, soil characteristics, and the temporal evolution of infiltration rate on vacant lots (likely a function of human activity and impact, topography, and vegetation).

As a simple representation for how we conceptualize the movement of water through vacant land areas, the model makes the following fundamental assumptions:

1) Vacant lots are gently sloped, thereby slowing the velocity of runoff at the surface and lateral water redistribution in the subsurface

2) Low infiltration rates were noted on lots with gentle slopes, such that localized overland flow is retained on the lot, and is not otherwise routed onto impervious surfaces, nor out of the lot,

3) Lots are well-vegetated, with vegetation providing resistance to the movement of water at the surface,

4) Groundwater table depth is well below the surface, diminishing the chance that runoff formation via saturation excess will occur, and

5) The near subsurface does not contain any restrictive layers that otherwise restrict redistribution of soil moisture and vertical percolation of infiltrated water.

These assumptions are explored in a few figures. To test for potential coincidence between low infiltration rates and high parcel slopes, conditions that would be expected to favor runoff generation, we compared average infiltration rate and parcel slope across 520 properties (Supplementary Figure 12). In general, vacant lots are flat, and few have slopes that exceed 2%. A small number of vacant lots had both infiltration rates < 0.1 cm hr^-1^ and steeper parcel slopes. In general, parcels with the slowest rates of infiltration (0.001 cm hr^-1^) occurred on parcels with gentle (<5%) topography. While infiltration rates in these areas will favor runoff generation, the lack of appreciable variety in topography likely kept runoff on the lot, and out of the local wastewater collection system. Therefore, rainfall detention capacity estimates may be underestimated.

We also explored the relationship between the fraction of each lot covered by vegetation and infiltration rate (Supplementary Figure 13). In general, most lots are vegetated, though these vegetated lots feature average infiltration rates that span three orders of magnitude.

To assess whether saturation overland flow may limit the potential of vacant lots to infiltrate precipitation, we gathered historical groundwater observations from across Buffalo. First, we queried the United States Geological Survey database to locate one-time observations of water table depth across the city (with the majority of the observations ca. 1960s and ca. 1980s). If multiple observations were taken at a location, we selected the observation closest to the surface. These water table depths are visualized in Supplementary Figure 7.

While many water table depths are several meters below the surface, four observations across the city, representing 17% of sites, suggest that the local water table is perched to within 0 to 0.3 m of the surface. Crucially, the majority of these water table observations are located in a low permeability lacustrine silt and clay surficial geology. These findings suggest that locally high water tables can occur across Buffalo (Supplementary Figure 7), but that many local water tables will still be relatively deep.

Though no timeseries observations of water table depths exist within the city, timeseries observations were available at a newly installed well (est. 2017) on the State University of New York Buffalo campus (Supplementary Figure 8). These observations suggest that the (local) water table remains at a relatively constant 6 meters depth below ground surface. Notably, this well is located in a till moraine, and expected to have greater permeability and therefore lower water tables than those in lacustrine silt and clay. Therefore, we expect vacant lots on the edges of the city to have low and consistent water tables that should not restrict any assumptions of free drainage.

**Supplementary Note 3:**

A complete evaluation of suitability and sustainability of vacant land as a resource for stormwater management should also consider its history – in particular, the potential contamination history – of the formerly occupied land. Across the City of Buffalo NY, the prior land use of vacant parcels was residential (Supplementary Table 11; 79.8% by number of parcels, 34.4% by land area). Although commercial and industrial properties constituted a smaller number of vacant parcels, these parcels tended to be much larger than residential properties, accounting for 32.2% (commercial) and 27.3% (industrial) of total vacant land.

Brownfields present with contaminated soils do occur across many cities, and Buffalo, NY is no exception. Though few vacant properties in Buffalo were formerly in industrial land use, disturbing these areas through demolition and changing their infiltration characteristics has potential to introduce contamination into local groundwater. Importantly, this underscores the need to investigate whether human management of stormwater via the introduction of green spaces (green infrastructure and vacant lots) may be turning a surface water contamination into groundwater contamination that we may be forced to contend with many years down the road. We are often challenged by the dearth of groundwater level measurements and water quality sampling; however, understanding surface characteristics, structure, and hydrological benefits within and across vacant lots is a first start down the path of teasing out pertinent groundwater information within the urban continuum.

**Supplementary Note 4:**

Vacant lots were assessed as per the site layout shown in Supplementary Figure 9, following the Vacant Land Assessment Protocol^2^. The protocol is based on nine station points (SPs), which were used to uniformly orient and standardize observations across each lot. Measurements were made by trained observers in 2016 and 2017, and included the following measurements listed below. A total of 718 parcels were analyzed with the full assessment protocol. Of these, there were 494 parcels where all observations were collected. We summarize the number and percentage of parcels with select observations across this dataset in Supplementary Table 12.

Soil textural class was estimated by feel^6^, with these nominal 50 g field moist soil samples taken adjacent to each of the two infiltration observation points. Soil textural class names are listed as taken from typical United States Department of Agriculture taxonomy. Results are shown in Supplementary Table 13.

To make assessments of soil penetration resistance (penetrometry, PE), assessors used a drop-hammer, single mass dynamic cone penetrometer to determine soil penetration resistance profiles at four locations per parcel. Nearly all lots include a PE assessment at SP9 (lot center). Assessors recorded the number of blows to reach standard depths of 5.1 cm, 10.2 cm, and 15.2 cm, or depth of refusal, and up to a maximum of 30 blows. Refusal is indicated by the data value 99. As there was not a standardized way to represent refusal, we present summary statistics in two ways – using a value of 99 to represent refusal (used during data collection; Figure 3) and using a value of 30 (maximum number of blows applied in protocol^2^; Supplementary Figure 3).

To determine within-parcel slopes, elevation was determined along four sight lines between the center of the lot (SP9) and each corner (SP1, SP2, SP3, SP4) using a stadia rod, sight, and hand level, then corrected for the height of the instrument (in other words, eye height of the assessor).

The proportion of vegetated cover for a parcel was recorded and expressed as a percentage, as was the proportion of impervious surfaces for a parcel. A higher resolution assessment of land cover was also performed, with land cover recorded at all nine station points using the following terminology: bare earth, vegetated other (trees or shrubs), vegetated grass, water (if standing water was present), concrete, and sealed soil, pavement, or structures.

Infiltration rate was measured twice in each lot with a Mini-Disk Portable Tension Infiltrometer (© Meter Group). One measurement was taken near the center of the lot (within the building footprint), and the other near the edge of the lot (outside the building footprint). While these observations were not standardized to station points, the latter measurement was typically conducted at SP2 or SP3, and the former was typically conducted near SP9 (roughly, what would constitute the backyard of a typical parcel). Prior to conducting infiltration tests, the land surface was cleared of vegetation to directly interface the infiltrometer with the soil surface, and the hydraulic tension head was set to -2 cm. Timeseries of infiltration volume were collected by reading and recording infiltrometer water level until a cumulative volume of at least 20 mL water was infiltrated, with measurements made at the following time increments: 0, 0.5, 1, 2, 3, 4, 5, 10, 15, 20, 30, 45, and 60 minutes. The level of effort specified for this measurement process was that the experiment was run either for a full hour or until at least 20 mL of water was infiltrated, as outlined in the Mini-Disk protocol^7^.

Infiltration rates were calculated from recorded volumes of infiltrated water through time and soil texture by fitting rates of cumulative infiltration against square root time marks, following:

$$I=C_{1}t+C_{2}\sqrt{t}$$

where ${C_{1}(}_{m}^{\propto}s^{-1}$) and ${C_{2} (m s}^{-1/2}$) represent parameters related to hydraulic conductivity, and soil sorptivity, respectively^7,8^. Infiltration rate, also referred to as near-saturated hydraulic conductivity, was calculated from the ratio of *C_1_* to a value calculated from van Genuchten parameters calculated for a given soil textural class^7,8^, the radius of the Mini Disk platform, and the suction at the disk surface. Soil textural class was based on tests made by feel^6^.

When the volume of infiltrated water is very slow, the curve fitting routine will yield negative infiltration rates, which was the case for eighteen parcels. To account for this, we replaced all negative values with a minimal, non-zero infiltration rate (0.001 cm hr^-1^). These parcels are essentially net producers of overland flow.

We incorporated uncertainty into infiltration estimates via an estimated error in volume readings, which was 1 mL (1 cm^3^). This estimate of uncertainty was added and subtracted from the volume read off at each time point, one at a time, for each infiltration run (e.g., at time points of 30, 60, 120, 180, 240, 300, 600, 900, 1200, 1800, 2700, and 3600 s) for a subset of infiltration rates (n = 426) with recorded timeseries of infiltrated volume. These values were propagated to produce two estimates of infiltration per parcel and were aggregated to develop a mean and 99% confidence intervals for each parcel (Supplementary Figure 10). As with our original estimates of infiltration rates, any negative values were set to a minimum estimate of 0.001 cm hr^-1^. Re-estimated infiltration rates are compared with measured infiltration rates in Supplementary Figure 11. The most pronounced differences occurred for sites with low infiltration rates (e.g., < 0.1 cm hr^-1^). While only 24 parcels within this subset of sites were measured to have very low rates of infiltration (0.001 cm hr^-1^), this number grew to 39 parcels when considering possible uncertainties in water level observations. These infiltration estimates are summarized for select sewersheds in Figure 1. To assess how uncertainty in infiltration rates may propagate into rainfall partitioning between runoff and infiltration across vacant lots, we estimated the fraction of precipitation estimated to infiltrate on a subset of vacant parcels (n = 426) for upper and lower bounds extracted from 99% confidence intervals. These bounds are shown in Figure 2.

We took several statistical approaches to assess whether infiltration rates were related to the year in which a parcel was demolished; or, in a separate analysis, if infiltration rate data was spatially-structured or dependent on location within the city. We used Moran’s I, a metric of spatial autocorrelation, to determine whether infiltration rates were spatially structured, for the entire infiltration dataset and for sets of parcels located in each of the five most vacant sewersheds. Moran’s I and corresponding p-values are reported in Supplementary Table 2 and 3. A value of 0 indicates a completely random data distribution, and a value of +1 indicates strong spatial structure in data. Our analysis indicates that there is weak spatial organization of infiltration rates, across surveyed properties in Buffalo, NY (USA).

To assess whether infiltration rates may be influenced by demolition date, we plotted probability density distributions obtained via kernel density estimation. Distributions summarize parcel-averaged infiltration rates for parcels demolished within a six-month window from 2001 through 2013 in Supplementary Figure 2.

In Supplementary Figure 2, average parcel infiltration rates through time are most frequently in the range between 0.1 and 1 cm hr^-1^, with little sample variance attributed to year of demolition. Linear (r^2^) and nonlinear (Spearman’s Rank) correlation coefficients between demolition date, in terms of the number of months since January 2001, and infiltration rate were weak (Supplementary Table 4), suggesting timing of demolition is not a strong predictor of infiltration rate. To verify that this was not influenced by scale differences between infiltration rates, we calculated the strength of linear and nonlinear relationships with respect to log-transformed infiltration.

**Supplementary References**

1. New York State Parcels (2019), Erie 2018 Tax Parcels. Available at: <http://gis.ny.gov/parcels/> (Accessed 20 Sept 2019).
2. US EPA Urban vacant land assessment protocol EPA 832-R-15-013 (2016). Available at: <https://www.epa.gov/sites/production/files/2016-08/documents/buffalo_ny_urban_vacant_land_assessment_protocol.pdf>. (Accessed 1 October 2018).
3. Network for Environment and Weather Applications (NEWA) Hourly Precipitation - Buffalo International Airport (2018). Available at: http://newa.cornell.edu/index.php?page=hourly-weather. (Accessed on 20 October 2018).
4. National Climatic Data Center (NCDC) Daily Precipitation – Buffalo International Airport (2018). Available at: https://www.ncdc.noaa.gov/cdo-web/datasets#GHCND. (Accessed on 20 October 2018).
5. Herrmann, D. L., Schifman, L. A., & Shuster, W. D. Widespread loss of intermediate soil horizons in urban landscapes. *Proceedings of the National Academy of Sciences*, **115**(26), 6751-6755 (2018).
6. Thien, S. J. Determining soil texture by the ‘‘feel method’’. *Journal of Agricultural Education* **8,** 54-55 (1979).
7. METER Group, Inc. Mini Disk Infiltrometer Manual (2018). Available at: http://manuals.decagon.com/Manuals/10564_Mini%20Disk%20Infiltrometer_Web.pdf. (Accessed 1 Oct 2018).
8. Zhang, R. (1997) Determination of soil sorptivity and hydraulic conductivity from the disk infiltrometer. *Soil Sci. Soc. Am. J.* **61**, 1024-1030.
9. Carsel, R. F., Parrish, R. S. Developing joint probability distributions of soil water retention characteristics. *Water Resour. Res.* **24**, 755-769 (1988).
